# An automatic segmentation method for heart sounds

**DOI:** 10.1186/s12938-018-0538-9

**Published:** 2018-08-06

**Authors:** Qingshu Liu, Xiaomei Wu, Xiaojing Ma

**Affiliations:** 1 Department of Electronic Engineering, Fudan University, Room 522 B, Science Building, 220 Handan Rd., Shanghai, China; 2 Key Laboratory of Medical Imaging Computing and Computer Assisted Intervention (MICCAI) of Shanghai, 138 Medical College Rd., Shanghai, China; 3 Children’s Hospital of Fudan University, 399 Wanyuan Rd., Shanghai, China

**Keywords:** Heart sound segmentation, Murmur elimination, Boundary detection, Component identification

## Abstract

**Background:**

There are two major challenges in automated heart sound analysis: segmentation and classification. An efficient segmentation is capable of providing valuable diagnostic information of patients. In addition, it is crucial for some feature-extraction based classification methods. Therefore, the segmentation of heart sound is of significant value.

**Methods:**

This paper presents an automatic heart sound segmentation method that combines the time-domain analysis, frequency-domain analysis and time–frequency-domain analysis. Employing this method, the boundaries of heart sound components are first located, and the components are then recognized. Finally, the heart sounds are divided into several segments on the basis of the results of boundary localization and component identification.

**Results:**

In order to evaluate the performance of the proposed method, quantitative experiments are performed on an authoritative heart sound database. The experimental results show that the boundary localization has a sensitivity (Se) of 100%, a positive predictive value (PPV) of 99.3% and an accuracy (Acc) of 99.93%. Moreover, the Se, PPV and Acc of component identification reach 98.63, 99.86 and 98.49%, respectively.

**Conclusion:**

The proposed method shows reliable performance on the segmentation of heart sounds. Compared with previous works, this method can be applied to not only normal heart sounds, but also the sounds with S3, S4 and murmurs, thus greatly increasing the applied range.

## Background

Cardiovascular disease (CAD) remains the leading cause of death worldwide. In China, CAD accounted for more than 40% of deaths in 2014 [1]. Heart sounds, which are generated by the beating of heart, are considered as an important signal for detecting cardiovascular problems. In general, a normal heart sound comprises two components, namely the first heart sound (S1) and the second heart sound (S2). In some special cases, three additional components, namely the third heart sound (S3), fourth heart sound (S4), and murmurs, may appear together or separately [2]. Heart sounds have been used to diagnose cardiovascular problems for hundreds of years. Even currently, auscultation remains a crucial approach for physicians to learn about patients’ heart health. However, this conventional diagnostic approach is highly subjective and relies largely on physicians’ experience [3]. Thus, an accurate and efficient automated heart sound analysis system is required.

There are two major challenges in developing an automated heart sound analysis tool: segmentation and classification. The primary operations of segmentation are positioning the boundaries of heart sound components and identifying the component types, namely S1, S2, S3, and S4. Several segmentation methods have been reported. However, most of the methods focus on either boundary detection or component identification. In addition, many methods only apply on normal heart sounds, thus severely limiting the application of these methods.

Liang et al. [4] proposed a Shannon-entropy-based heart sound segmentation method to recognize S1 and S2, but they did not study S3, S4, and murmurs. Kumar et al. [5] presented an S3 detection algorithm based on wavelet transform; however, this method could not detect the boundaries of the heart sound components. Moukadem et al. [6] developed an S-transform-based heart sound segmentation method; however, this method can only be applied to normal heart sounds. Springer et al. [7] successfully addressed the segmentation problem of noisy S1 and S2 by utilizing a logistic regression–hidden semi-Markov model. Nevertheless, this study, as most other studies, did not address the segmentation of heart sounds containing S3 and S4. In addition to the aforementioned studies, several other researchers have also contributed to the automated segmentation of heart sounds. For example, Naseri et al. [8] proposed a heart sound segmentation method based on the frequency-energy method, which had excellent performance for various heart sounds. Boutana et al. [9] presented a time–frequency-analysis–based heart sound segmentation method and validated the efficiency of the method through some pathological heart sounds. Tang et al. [10] reported a dynamic-clustering-based segmentation method, which was effective for both normal and abnormal heart sounds. However, these methods all have some of the aforementioned limitations.

Based on the advantages and drawbacks of the methods mentioned above, the present study is focused on developing an automated heart sound segmentation algorithm that can position the boundaries and recognize the components for both normal heart sounds and heart sounds with S3, S4 and murmurs.

**Fig. 1:**
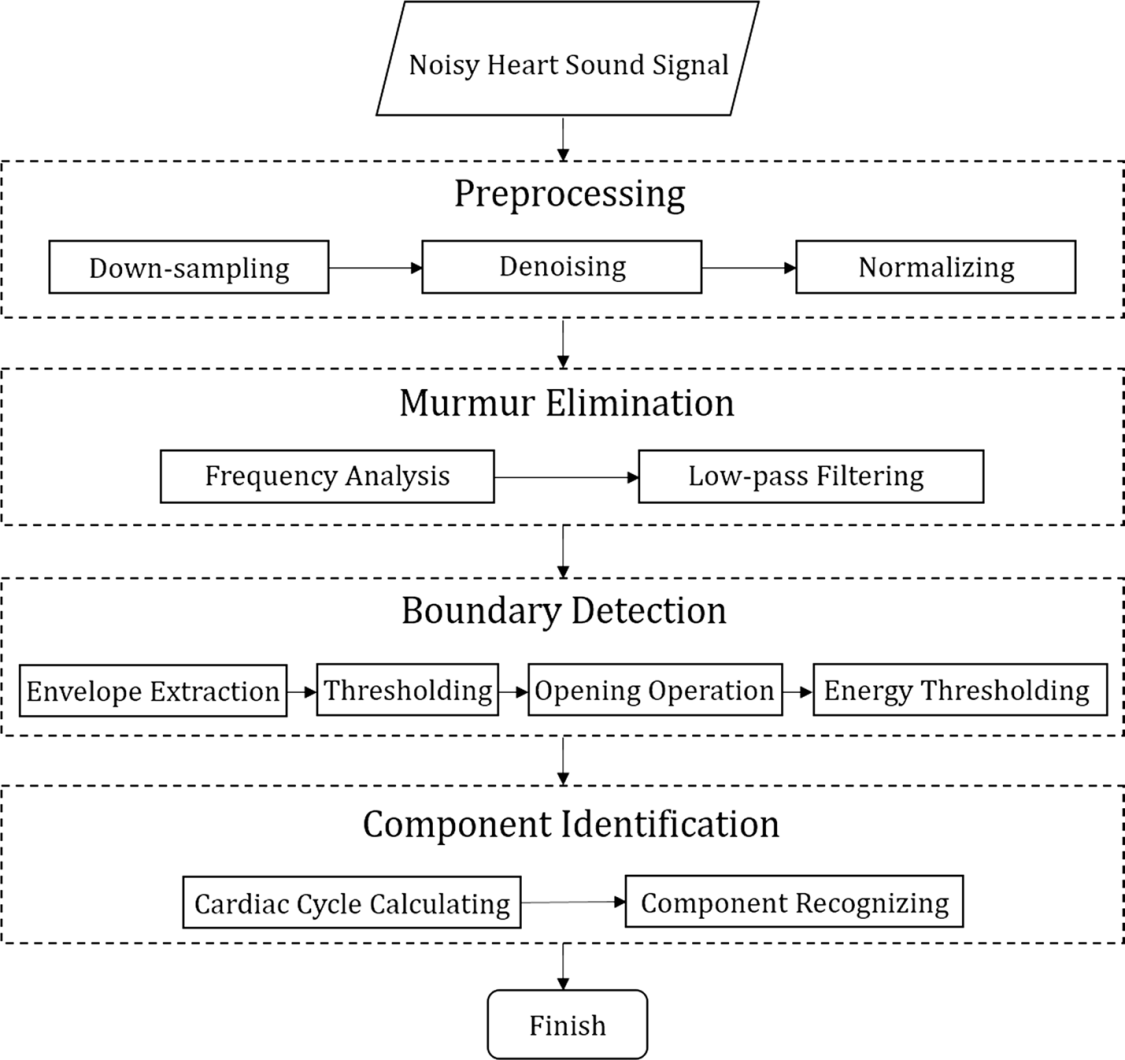
Block diagram of the proposed method

In this study, a method that combines time-domain analysis, frequency-domain analysis, and time–frequency-domain analysis was developed, and some novel strategies were proposed. The complete method comprises four parts, namely preprocessing, murmur elimination, boundary detection, and component identification. The overall process is presented in Fig. 1. First, the noisy raw heart sound is standardized in the preprocessing phase. Then, the signal goes through murmur elimination algorithm, where any possible murmurs are considerably eliminated without affecting the characteristics of the heart sound signal. In the following boundary detection phase, the onset and offset of each heart sound component are positioned and marked automatically. Finally, in the component identification phase, the cardiac cycle is calculated first, and the heart sound components are then recognized in each heartbeat on the basis of cardiac cycle.

## Methods

### Materials

To validate the proposed method, two quantitative experiments were performed, and the heart sounds from the University of Michigan’s Heart Sound & Murmur Library were employed [11]. In the first experiment, 12 types of heart sounds with variable murmurs were used to verify the effect of murmur elimination. The total length of these sounds was 759 s, and the cardiac cycle number reached 888. The second experiment was performed to quantify the effectiveness of boundary detection and component identification. For this purpose, 16 types of sounds, including 2 normal and 14 abnormal types, were used, constituting a total of 1039 s (1251 cycles). The heart sound signals were recorded at 44.1 kHz, and each one lasted for at least 56 s. For the convenience of displaying and testing, each sound was divided into three-heart-cycle segments. Moreover, every two adjacent segments had an overlap of one cardiac cycle.

### Preprocessing

#### Down-sampling

Because the maximal frequency of the heart sounds did not exceed 1 kHz [12], the heart sound signals were down-sampled to 2 kHz according to the Nyquist-Shannon sampling theorem.

#### Denoising

A wavelet-based denoising method with soft threshold was utilized. The db4 wavelet was chosen as mother wavelet because its shape is similar to that of a heart sound signal, and the scale was established as 7 based on signal–noise-ratio (SNR) and normalized root mean squared error (NRMSE) [13]. The denoised heart sound was written as $HS\left( n \right)$.

#### Normalization

To standardize the processing of heart sound, the heart sound signals were normalized as

1 $$HS_{N} \left( n \right) = \frac{HS\left( n \right)}{{{ \hbox{max} }\left( {\left| {HS\left( n \right)} \right|} \right)}} , \quad n = 0,1, \ldots ,N - 1$$

where N is the total number of sampling points.

Unless otherwise specified, the expressions “original heart sound” and “original signal” appearing in the following refer to *HS*_*N*_(*n*).

### Murmur elimination

Different from the conventional constant-cutoff-frequency-based murmur elimination method (usually 200 Hz) [8, 14], this paper presents a novel low-pass filter to remove the murmurs, namely the automatic-cutoff-frequency low pass filter (ALPF), whose cutoff frequency is calculated by analyzing the fast Fourier transform (FFT) of the heart sound.

Assuming that the modulus of the FFT sequence of $HS_{N} \left( n \right)$ is $FFT_{H} \left( n \right)$, *n* = 0, 1, …, *N *− 1, the envelope of $FFT_{H} \left( n \right)$ is obtained by the moving average method as follows:

$$E_{FFT} \left( n \right) = \frac{1}{{L_{F} + n + 1}}\sum\nolimits_{k = 0}^{{n + L_{F} }} {FFT_{H} \left( k \right)} ,\quad 0 \le n \le L_{F} - 1$$

2 $$E_{FFT} \left( n \right) = \frac{1}{{2L_{F} + 1}}\sum\nolimits_{{k = n - L_{F} }}^{{n + L_{F} }} {FFT_{H} \left( k \right)} , \quad L_{F} \le n \le N - L_{F} - 1$$

$$E_{FFT} \left( n \right) = \frac{1}{{L_{F} + N - n}}\sum\nolimits_{{k = n - L_{F} }}^{{n + L_{F} }} {FFT_{H} \left( k \right)} ,\quad N - L_{F} \le n \le N - 1$$

where N is the sampling number of *FFT*_*H*_(*n*), and *L*_*F*_ is the neighborhood radius of point n, *L*_*F*_ ≪ N. Note that if *L*_*F*_ is too large, some adjacent local peaks of $E_{FFT} \left( n \right)$ are merged together. Considering this and experimental results, *L*_*F*_ is set to 5. The envelope is also normalized using (1) and is written as $E_{F} \left( n \right)$. Unless otherwise specified, the “FFT coefficient” and “FFT envelope” appearing hereinafter refer to $E_{F} \left( n \right)$.

**Fig. 2:**
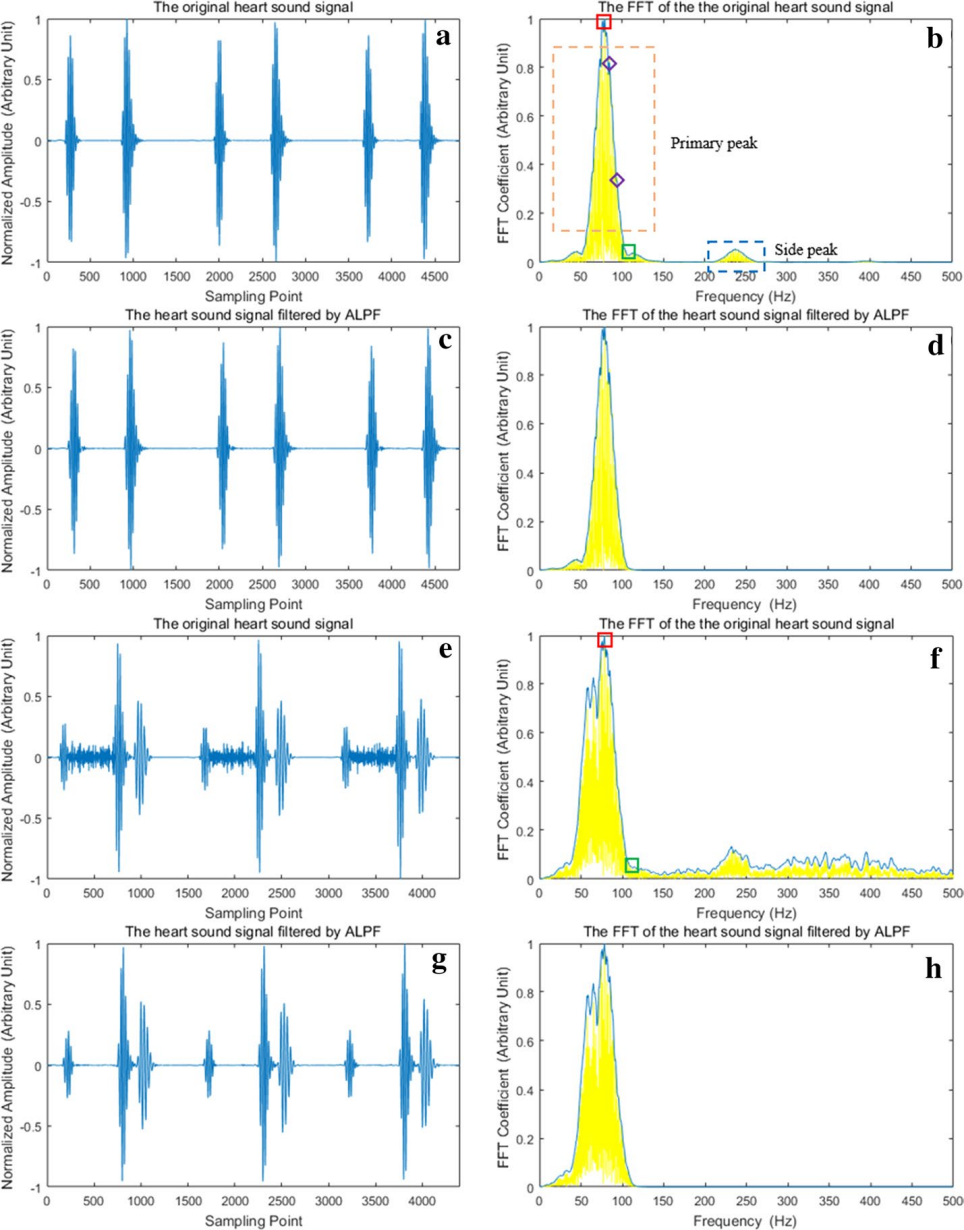
The performance of ALFP. **a** Is a normal heart sound, **c** is the filtered normal sound by ALFP, **e** is a heart sound with S3 and systolic murmurs, **g** is the murmur-eliminated sound by ALPF. **b**, **d**, **f** and **h** Are the FFT of **a**, **c**, **e** and **g**, respectively. In **b**, **d**, **f** and **h**, the yellow curves are the FFT sequences, and the blue curves are the envelopes of them. In **b**, the points marked by purple diamonds are two examples of spikes. In **b** and **f**, the points marked by red squares and green squares are the primary peak points and the last valley points with FFT coefficient smaller than 0.2, respectively

For the heart sounds without murmurs,$E_{F} \left( n \right)$ is generally comprised of primary peak and side peak (see in Fig. 2b). The primary peak is located in the low-frequency area with the highest amplitude, and the side peak is located in the relatively higher-frequency area with much smaller amplitude. Thus, most energy of heart sounds is concentrated within the frequencies of primary peak, and filtering out the side peak does not affect the shape of heart sounds (see in Fig. 2c, d). Therefore, the primary peak preserves the information of heart sound components (S1, S2, S3 and S4).

For the heart sounds with murmurs, the primary peak still exists, but the side peak is usually merged with the frequency band of murmurs (see in Fig. 2f). Considering that the murmurs usually have higher frequency than the primary peak, the frequency of primary peak’s ending point is an appropriate estimation of the cutoff frequency to remove murmurs.

In this study, the ending point of primary peak is searched in the range of 20 to 200 Hz and is determined as the first valley point (the lowest point between two adjacent spikes) after the primary peak point with FFT coefficient smaller than 0.2. The value 0.2 is determined based on experimental experience. If there is no such an ending point between 20 and 200 Hz, the cutoff frequency is set to 200 Hz.

$HS_{N} \left( n \right)$ is filtered using this frequency, and the filtered sound is then normalized; the normalized sound is denoted as *HS*_*NLP*_.

### Boundary detection

#### Envelope extraction

After removing the interference of murmurs, the next step is to calculate the onsets and offsets of heart sound components. Because the envelope can reduce the complexity of computing while preserving the location information of the signals, the closing operation of mathematical morphology is utilized to obtain the envelope in the proposed method and is defined as

3 $$\left[ {f \cdot g} \right]\left( n \right) = \left\{ {\left[ {f \oplus g} \right] \otimes g} \right\}\left( n \right)$$

where *f*(*n*) refers to the input signal, and *g*(*n*) is a structure element; ⨁ denotes the dilation operation, and ⨂ denotes the erosion operation. The lengths of *f*(*n*) and *g*(*n*) are P and Q respectively, and generally P > Q [15, 16].

The choice of structure element *g*(*n*) directly affects the shape of the envelope obtained by this operation [17]. In this study, in order to make the shape of envelope simple, *g*(*n*) is designed as

4 $$g\left( n \right) = 0, \quad 0 \le n \le Q$$

Moreover, Q, the length of $g\left( n \right)$, is also a significant parameter. According to physiological knowledge, in general, the time interval between adjacent heart sound components (S1S2 interval, S2S3 interval, S4S1 interval, etc.) is no less than 100 ms [8, 18], which corresponds to 200 points under the sampling rate of 2 kHz. Thus, Q should be smaller than 200. In this study, Q was set as 30 on the basis of the experimental results.

The $f\left( n \right)$ in (3) is replaced by *HS*_*NLP*_, and the heart sound envelope is obtained. The envelope is then normalized. The result is denoted as *E*_*NLP*_.

Although the ALPF can effectively reduce murmurs, the filtered heart sound may retain some low-amplitude residuals, which are also reflected in the envelope and affect the detection of boundaries. In this method, three operations are used to overcome this problem.

#### Thresholding processing

The first operation is thresholding.

For some murmurs, such as early systolic murmurs, their onsets partially overlap the offsets of S1s. After filtering by ALPF, the residues of murmurs may still be linked with S1s. Consequently, the murmur residues and S1s may be mixed together in $E_{NLP}$. Therefore, the purpose of thresholding is to separate the boundaries of murmurs from those of S1s to conduct the subsequent operations. Consequently, a small threshold can complete this task. Due to the use of small threshold, the boundary information of the heart sound components can be preserved as much as possible.

The envelope $\tilde{E}_{NLP}$ after thresholding is calculated as

5 $$\tilde{E}_{NLP} = \left\{ {\begin{array}{ll} E_{NLP} ,& \quad E_{NLP} \ge \theta \\ 0,&\quad E_{NLP} < \theta \\ \end{array} } \right.$$

where *θ* = min(*θ*_*A*_, *θ*_*C*_), $\theta_{A} = \lambda \cdot \sqrt {\frac{1}{N}\mathop \sum \nolimits_{n = 0}^{N - 1} \left[ {E_{NLP} \left( n \right) - \mu } \right]^{2} }$, $\lambda = 0.8, \mu = \frac{1}{N}\mathop \sum \nolimits_{m = 0}^{N - 1} E_{NLP} \left( m \right)$ and *θ*_*C*_ = 0.025. The parameter *θ*_*A*_ enables the algorithm to determine an appropriate threshold based on the characteristics of the signal itself, and *θ*_*C*_ is a reference threshold, preventing error in *θ*_*A*_; the value of *θ*_*C*_ is determined by experimental results.

#### Opening operation

After thresholding, the boundaries of heart sound components and interfering residues are separated. The remaining residues are considerably smaller than the heart sound components in both duration and amplitude. Because the opening operation of mathematical morphology can reduce spikes, it is utilized as the second stage of residue removal. The opening operation is defined as [19]

6 $$\left[ {f \circ g} \right]\left( n \right) = \left\{ {\left[ {f \otimes g} \right] \oplus g} \right\}\left( n \right)$$

The *g*(*n*) in opening operation is still defined using (4), but the length of *g*(*n*) is denoted as Q’. Besides, the parameters in (6) are same as those in (3).

As in the closing operation, Q’ is also a significant parameter. Because the average durations of S1 and S2 are approximately 100 ms and the durations of S3 and S4 are approximately 50 ms [8], which correspond to 200 and 100 sampling points, respectively. Thus, Q’ must be smaller than 100; in this study, it was set to 50 based on experimental results. The results of the opening operation are normalized, and the normalized signal is denoted as $\tilde{E}_{NO}$.

#### Energy thresholding processing

Finally, to completely exclude the interference components from the envelope, the energy thresholding approach is used. Considering that $\tilde{E}_{NO}$ is comprised of points of zero and non-zero, i.e., the regions located by S1, S2, S3, S4 and few murmur residues are non-zero, and the rest regions are zero. Therefore, the boundaries of each component (including the interference components escaping the opening operation) can be automatically obtained as follows:

$$If \tilde{E}_{NO} \left( i \right) = = 0 \& \& \tilde{E}_{NO} \left( {i + 1} \right) \ne 0,\quad i = 0,1, \ldots N - 2$$

then, the *i*th point is determined as a potential onset;

$$If \tilde{E}_{NO} \left( {j - 1} \right) \ne 0 \& \& \tilde{E}_{NO} \left( j \right) = = 0,\quad j = 1,2, \ldots N - 1$$

the *j*th point is determined as a potential offset. The potential onset and the potential offset always appear in pairs and these onsets and offsets are denoted as *SPT*(*i*) and *EPT*(*j*) respectively, where $i, j = 0,1, \ldots ,M - 1$. *M* is the number of potential onsets/offset.

Consequently, the energy of each component can be obtained as

7 $$E_{k} = \sum\nolimits_{i = SPT\left( k \right)}^{EPT\left( k \right)} {\tilde{E}_{NO} {}^{2}\left( i \right)} ,\quad k = 0,1, \ldots ,M - 1$$

Assuming for each *E*_*k*_,

$$If \ E_{k} < \eta$$

then the *k*th potential onset and offset are considered invalid and removed from $SPT$ and *EPT*. Moreover, the points between these invalid onsets and offsets are determined as interference parts and are set to zero using (8)

8 $$\tilde{E}_{NO} \left( n \right) = \left\{ {\begin{array}{l} {0,\ {SPT}\left( k \right) \le n \le EPT\left( k \right), \ 0 \le k \le M - 1, \ if \ E_{k} < \eta } \\ {\tilde{E}_{NO} \left( n \right), \ else } \\ \end{array} } \right.$$

In this inequality, $\eta$ is set to 0.25 based on experimental experience. The remaining potential points are determined as the final onsets and offsets of the heart sound components.

### Component identification

#### Cardiac cycle calculation

The last step is to recognize the heart sound components. Considering the quasi-periodic nature of heart sounds, this step can be more efficiently accomplished if the cardiac cycle is calculated. In some studies, the cardiac cycle was calculated by using the partial autocorrelation function (PACF) [10, 15]. However, because of the inherent defects of PACF, the calculating results are not satisfactory. In order to overcome the shortcomings of PACF, this study proposes a cardiac cycle calculation method based on the unbiased autocorrelation function (UACF), considerably improving the applicability.

**Fig. 3:**
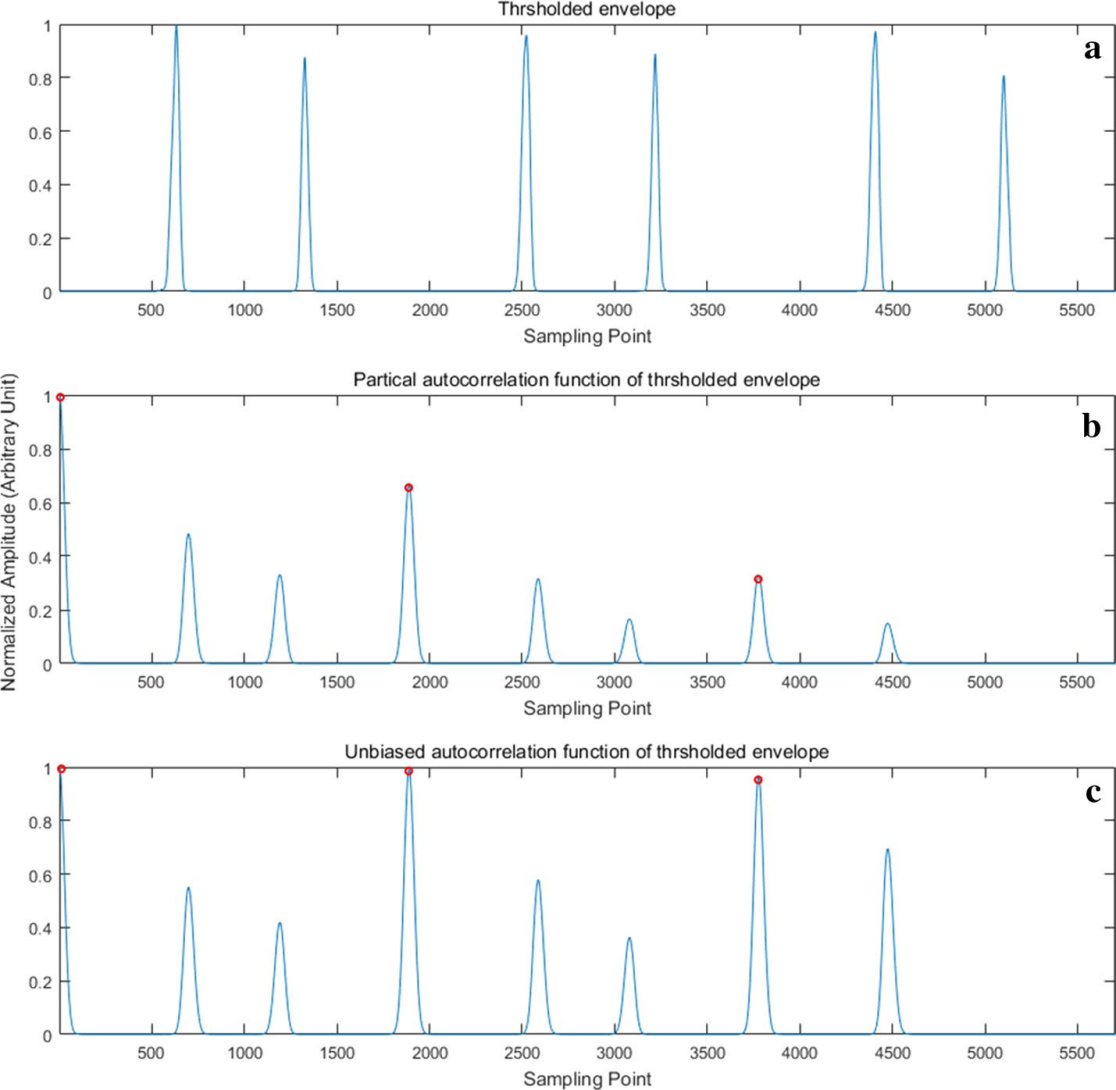
PACF and UACF of heart sound: **a** is the thresholded heart sound envelope $\tilde{E}_{NO}$; **b** is the PACF of **a**; **c** is the UACF of **a**. The peaks that are marked by red circles are primary peaks, while the peaks unmarked are side peaks

The PACF and UACF are defined as (9) and (10), respectively.

9 $$R\left( m \right) = \frac{1}{N}\sum\nolimits_{n = 0}^{N - m - 1} {\tilde{E}_{NO} \left( n \right)\tilde{E}_{NO} \left( {n + m} \right)}$$

10 $$R^{\prime}\left( m \right) = \frac{1}{N - m}\sum\nolimits_{n = 0}^{N - m - 1} {\tilde{E}_{NO} \left( n \right)\tilde{E}_{NO} \left( {n + m} \right)}$$

where $m = 0,1, \ldots N - 1, N$ is the sampling number of $\tilde{E}_{NO}$. Figure 3 demonstrates the PACF and UACF.

For PACF, as m increases, the number of sampling points involved in multiplication and accumulation decreases gradually. Consequently, the primary peaks of the PACF exhibit gradual attenuation (see in Fig. 3b), causing the side peaks between the primary peaks to be interference of the cardiac cycle calculation. By contrast, the UACF only averages the terms that are involved in multiplication and accumulation, thereby overcoming the drawback of primary peak’s attenuation in the PACF.

Figure 3c shows that there is a relatively significant difference in amplitude between the primary peaks and side peaks. To further expand the amplitude difference, square energy, which is defined in (11), is utilized. After obtaining the energy signal, thresholding is used to remove the side peaks. The energy signal of the UACF and the energy signal after thresholding are denoted as $E_{{R^{\prime}}}$ and $\tilde{E}_{{R^{\prime}}}$, respectively.

11 $$E_{{R^{\prime}}} \left( m \right) = R^{{\prime}^{2}} \left( m \right),\quad m = 0,2, \ldots, N - 1$$

12 $$\tilde{E}_{{R^{\prime}}} = \left\{ {\begin{array}{ll} E_{{R^{\prime}}} ,&\quad E_{{R^{\prime}}} \ge \sigma \\ 0,&\quad E_{{R^{\prime}}} < \sigma \\ \end{array} } \right.$$

where $$\sigma = 0.4 \times { \hbox{max} }\left( {E_{{R^{\prime}}} } \right)$$.

**Fig. 4:**
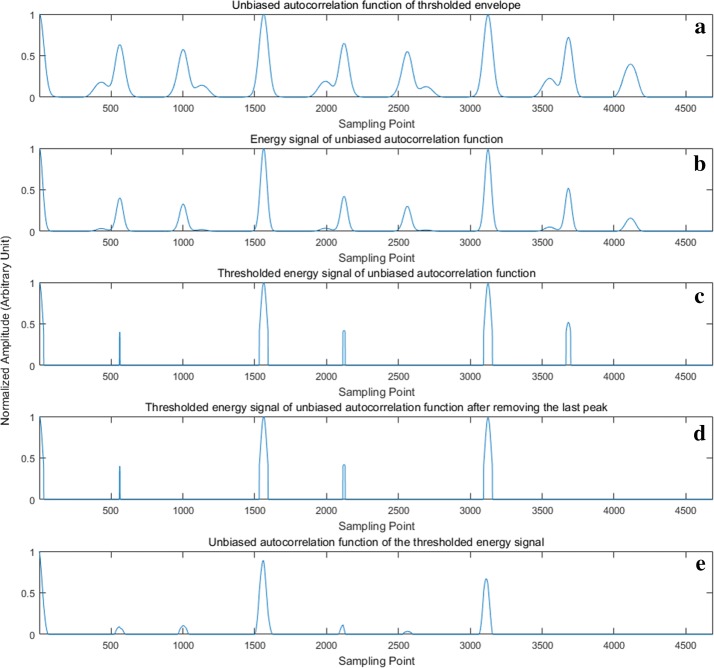
The main procedures of cardiac cycle calculation: **a** is *R*^′^(*m*), the UACF of a heart sound with S4; **b** is $E_{{R^{\prime}}} \left( m \right)$, the energy signal of *R*^′^(*m*); **c** is $\tilde{E}_{{R^{\prime}}} \left( m \right)$; **d** is **c** without the last peak; **e** is *R*_*Final*_, the UACF of **d**

The last peak of $\tilde{E}_{{R^{\prime}}}$ is likely to be a side peak that escaped from thresholding because of its relatively higher amplitude (see in Fig. 4c). This kind of side peak is usually generated by the multiplication and accumulation of S1 and S2. In order to calculate the cardiac cycle accurately, the last peak of $\tilde{E}_{{R^{\prime}}}$ is forcibly removed regardless of whether it is a side peak or a primary peak (see in Fig. 4d). Finally, the UACF of $\tilde{E}_{{R^{\prime}}}$ with the last peak removed is calculated, and this UACF is denoted as *R*_*Final*_ (see in Fig. 4e). Then, thresholding is performed on *R*_*Final*_ with a threshold of 0.5 × max (*R*_*Final*_), leaving only primary peaks in the thresholded *R*_*Final*_. The intervals between adjacent peaks of thresholded *R*_*Final*_ are then calculated and averaged to obtain the average cardiac cycle. Figure 4 shows the main procedures of cardiac cycle calculation.

#### Component recognition

After determining the cardiac cycle, the heart sound components can be recognized beat by beat. Under normal circumstances, in terms of the number of components in one heartbeat, heart sounds can be divided into three categories: two components (S1 and S2), three components (S1, S2, S3, or S4) and four components (S1, S2, S3, and S4). Moreover, in the time domain, S1S2 interval < S2S1 interval, S2S3 interval < S3S1 interval, and S2S4 interval > S4S1 interval, whereas in the frequency domain, the frequencies of S1 and S2 are usually higher than those of S3 and S4 [8, 15]. These priori knowledge along with the mentioned three categories can be utilized to recognize the heart sound components.

**Fig. 5:**
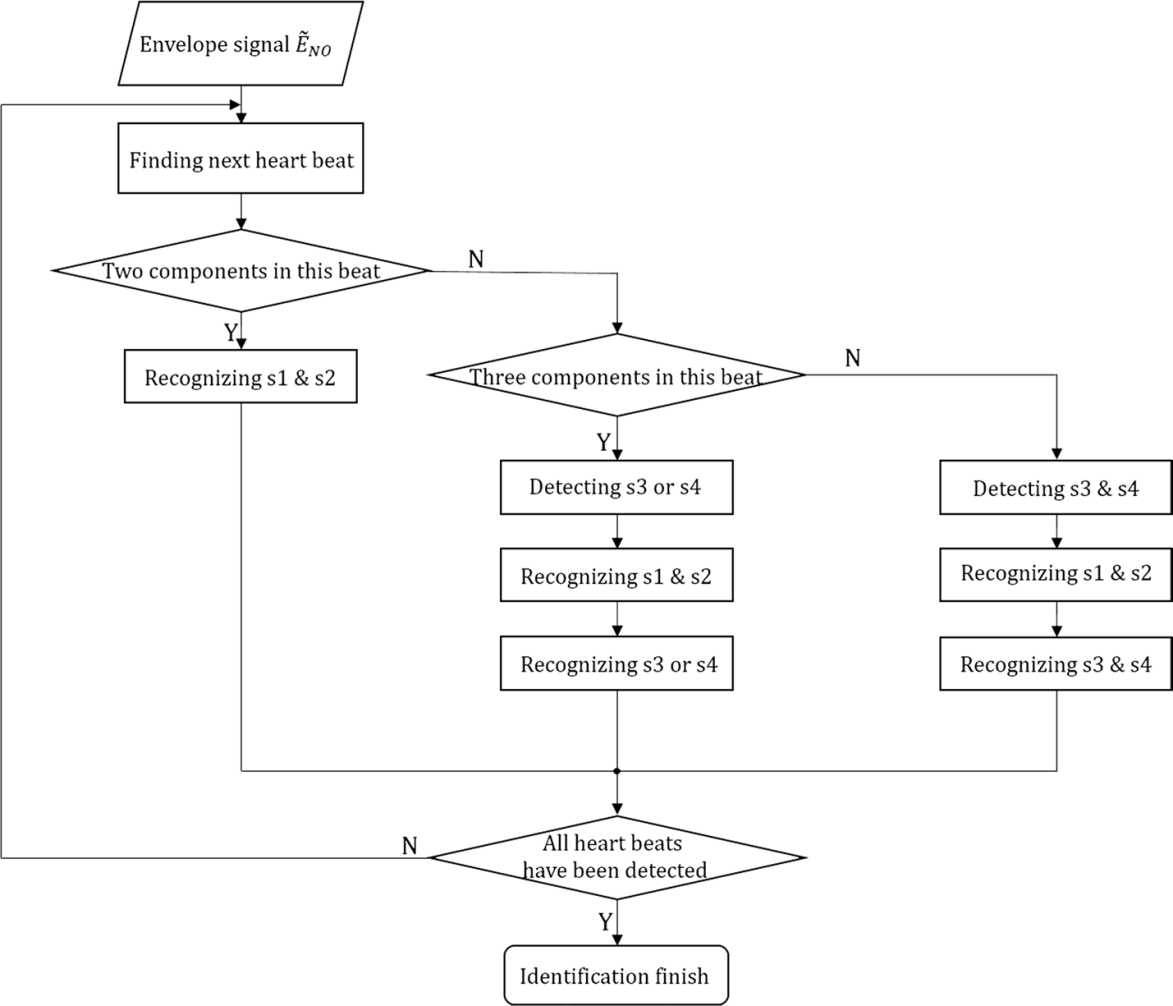
Flow diagram of heart sound component identification

Figure 5 presents the overall process of component recognition. The algorithm finds a heartbeat based on cardiac cycle and automatically obtains its component number. Then, the recognition processing is performed according to the component number found.

For the convenience of expression, in the following part, the *j*th component of the *i*th heartbeat is denoted as *C*_*j*_^*i*^, and the time interval between *C*_*m*_^*p*^ and *C*_*n*_^*q*^ is denoted as *T*_*mn*_^*p*, *q*^.

Assuming that the $i$ th heartbeat is being analyzed, then the recognition processing can be expressed as follows.

Two components: In this case, *T*_12_^*i*, *i*^ and *T*_21_^*i*, *i*+1^ are calculated. If *T*_12_^*i*, *i*^< *T*_21_^*i*, *i*+1^, *C*_1_^*i*^is identified as S1, and *C*_2_^*j*^ is identified as S2; otherwise, *C*_1_^*i*^is S2, and *C*_2_^*j*^ is S1.

Three components: In this case, because S3 and S4 are less than S1 and S2 in frequency, these three components can be recognized using three steps: detecting S3/S4, recognizing S1 and S2, and identifying S3/S4.

Time–frequency analysis is required for the detection of S3 and/or S4. In this study, S-transform is employed to accomplish this task. Assuming that the signal to be analyzed is *h*(*n*), *n* = 0, 1, …, *N *− 1, its Fourier transform is $H\left( {k/NT} \right),k = 0,1, \ldots ,N - 1,$ where T is the sampling interval. Then, the S-transform of $h\left( n \right)$ is given by [20].

13 $$\left\{ {\begin{array}{l} {S\left( {jT,\frac{n}{NT}} \right) = \sum\nolimits_{m = 0}^{N - 1} {H\left( {\frac{m + n}{NT}} \right)e^{{ - \frac{{2\pi^{2} m^{2} }}{{n^{2} }}}} e^{{\frac{i2\pi mj}{N}}} } ,\quad n \ne 0} \\ {S\left( {jT,0} \right) = \frac{1}{N}\sum\nolimits_{m = 0}^{N - 1} {\left( {\frac{m}{NT}} \right)} ,\quad n = 0} \\ \end{array} } \right.$$

where *j*, *m*, *n* = 0, 1, …, *N *− 1.

To obtain sufficient time–frequency information, the S-transform is directly performed on the original heart sound signal *HS*_*N*_(*n*). The result is a complex matrix and is written as *S*. Suppose that the element of the *m*th row in the *n*th column of the matrix is *S*_*m*,*n*_ = *a* + *jb*, then the modulus of $S_{m,n}$ is defined as.

14 $$\left| S \right|_{m,n} = |S_{m,n} | = \sqrt {a^{2} + b^{2} }$$

The modulus of each element of *S* is calculated, and the modulus matrix |*S*| is then obtained. Unless otherwise specified, both the expression “S transform” and “S coefficient” in the following text refer to $\left| S \right|$.

The two-dimensional |*S*| matrix is relatively difficult to analyze; thus, the one-dimensional instantaneous frequency is utilized to detect S3 and S4, and is defined as.

15 $$f_{H} \left( n \right) = \frac{{\mathop \sum \nolimits_{m = 0}^{N - 1} F\left( m \right) \cdot \left| S \right|_{m,n} }}{{\mathop \sum \nolimits_{m = 0}^{N - 1} \left| S \right|_{m,n} }}$$

where $F\left( m \right) = \frac{m}{NT} = \frac{{m \cdot f_{s} }}{N}$ represents the frequency of the *m*th row of the S-transform, and *f*_*s*_ is the sampling rate. Because the maximal frequency of the sampled signal is half of the sampling frequency, $F\left( m \right)$ was set as $\frac{1}{2}\frac{m}{NT} = \frac{{m \cdot f_{s} }}{2N},m = 0,1, \ldots ,N - 1$ in this study.

**Fig. 6:**
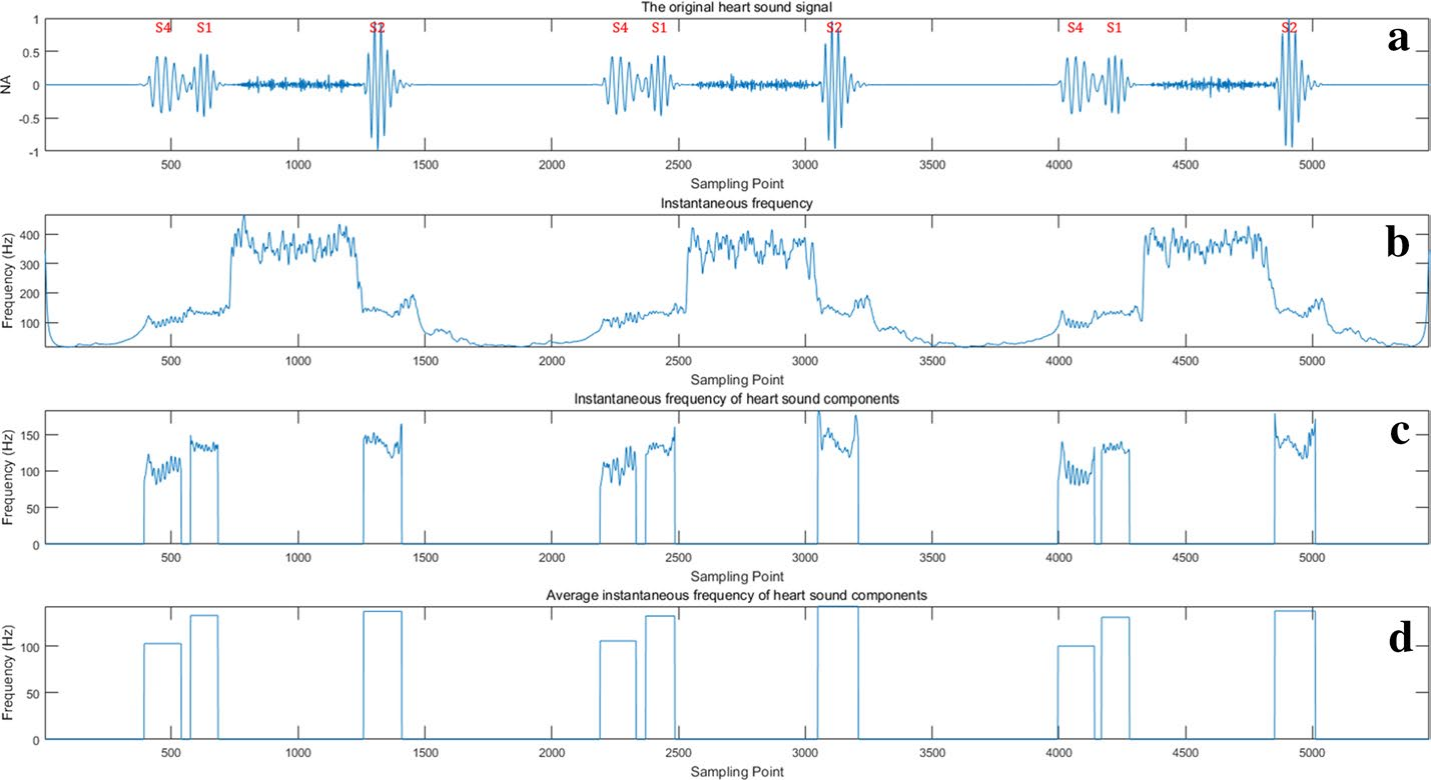
Time–frequency analysis: **a** is a heart sound with murmurs and S4; **b** is the instantaneous frequency of this heart sound; **c** is the instantaneous frequency only for the heart sound components; **d** is the average instantaneous frequency of **c**. “NA” represents “normalized amplitude”

Furthermore, the boundaries of heart sound components are obtained in the boundary detection phase (i.e., *SPT* and *EPT*). Therefore, by extracting the parts between these onsets and offsets from *f*_*H*_(*n*), the instantaneous frequency of heart sound components is obtained (see in Fig. 6(3)).

Finally, the average instantaneous frequency is calculated. Figure 6a–d present an example of these operations. It is observed that the average instantaneous frequency of S4 is significantly lower than those of S1 and S2. Similarly, the instantaneous frequency of S3 is also the lowest among S1, S2 and S3. Consequently, the component with the lowest frequency of each heartbeat is S3 or S4, which means that the previous component is S2 and the next is S1.

Without loss of generality, assuming that *C*_2_^*i*^ is the component with the lowest frequency, *C*_1_^*i*^ and *C*_3_^*i*^ can be easily determined as S2 and S1, respectively. Then, *T*_12_^*i*, *i*^ and *T*_23_^*i*, *i*^ are calculated and compared. If *T*_12_^*i*, *i*^ < *T*_23_^*i*, *i*^, *C*_2_^*i*^ is recognized as S4; otherwise, if *T*_12_^*i*, *i*^ > *T*_23_^*i*, *i*^, *C*_2_^*i*^ is S3.

Four components: In this case, the four components are arranged in the order: S1 → S2 → S3 → S4 → ··· S1 → S2. Thus, the first one of the two components with the lowest instantaneous frequencies is S3, and the second is S4. Moreover, the component preceding the detected S3 and S4 is S2, and the following component is S1.

After all the components in the *i*th heartbeat are recognized, the algorithm finds the next beat, and performs the same recognition processing. When all the heartbeats are analyzed, the recognition process is finished.

Finally, the heart sounds are divided into segments. For the sounds containing S1 and S2 only (including the sounds with murmurs), they are segmented into S1, S1S2 interval, S2 and S2S1 interval. For the sounds containing extra S3/S4 (sounds with murmurs are also included), they are segmented into S1, S1S2 interval, S2, S2S3/S2S4 intervals, S3/S4, S3S1/S4S1 intervals.

## Results

### Experimental setup

#### Murmur elimination evaluation

**Fig. 7:**
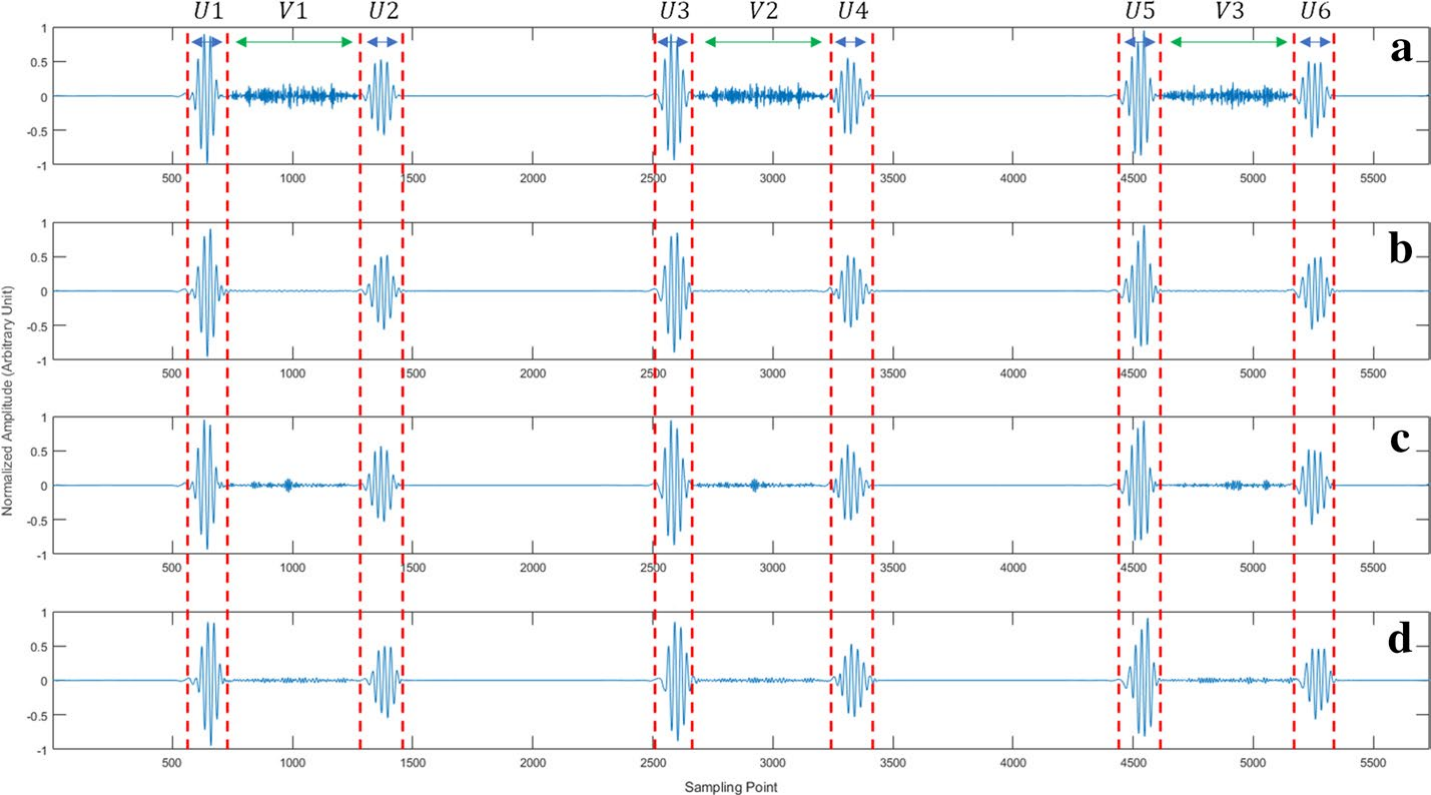
Murmur elimination effect for heart sound containing S4 and systolic murmurs using the three methods. **a** Is the non-murmur-reduced heart sound, and **b**–**d** show the sounds filtered by ALPF, WT and 200-LPF, respectively. In the calculation of $SMR, U = U1 + U2 + \cdot\cdot\cdot + U6$, and $V = V1 + V2 + V3$

For the evaluation of murmur elimination, a novel index, the signal murmur ratio (SMR), is proposed as follows:

16 $$SMR = 10 \cdot lg\frac{{\mathop \sum \nolimits_{n \in U} X^{2} \left( n \right)}}{{\mathop \sum \nolimits_{m \in V} X^{2} \left( m \right)}}$$

where *X*(*n*) is the heart sound signal, $U$ is the region where heart sound components (S1, S2, S3, and S4) are located, and *V* is the region where murmurs are located. To ensure the validity of the indicator, both *U* and $V$ are manually determined from the original signal *HS*_*N*_(*n*). Thereby, for a certain heart sound, *U* and *V* are constant when calculating SMRs of the murmur-reduced heart sound and the non-murmur-reduced heart sound (see in Fig. 7). A higher SMR indicates a smaller murmur energy proportion in signal $X\left( n \right)$, which means a stronger murmur removing effect.

#### Boundary detection and component identification evaluation

To validate the performance of boundary detection and component identification, three classical metrics, sensitivity (Se), positive predictive value (PPV), and accuracy (Acc) are calculated. These three indices are defined as follows:

17 $${\text{Sensitivity}}\left( {\text{Se}} \right) = \frac{\text{TP}}{{{\text{TP}} + {\text{FN}}}} \times 100{\text{\% }}$$

18 $${\text{Positive predictive value }}\left( {\text{PP}} \right) = \frac{\text{TP}}{{{\text{TP}} + {\text{FP}}}} \times 100{\text{\% }}$$

19 $${\text{Accuracy }}\left( {\text{Acc}} \right) = \frac{{{\text{TP}} + {\text{TN}}}}{{{\text{TP}} + {\text{TN}} + {\text{FP}} + {\text{FN}}}} \times 100{\text{\% }}$$

**Fig. 8:**
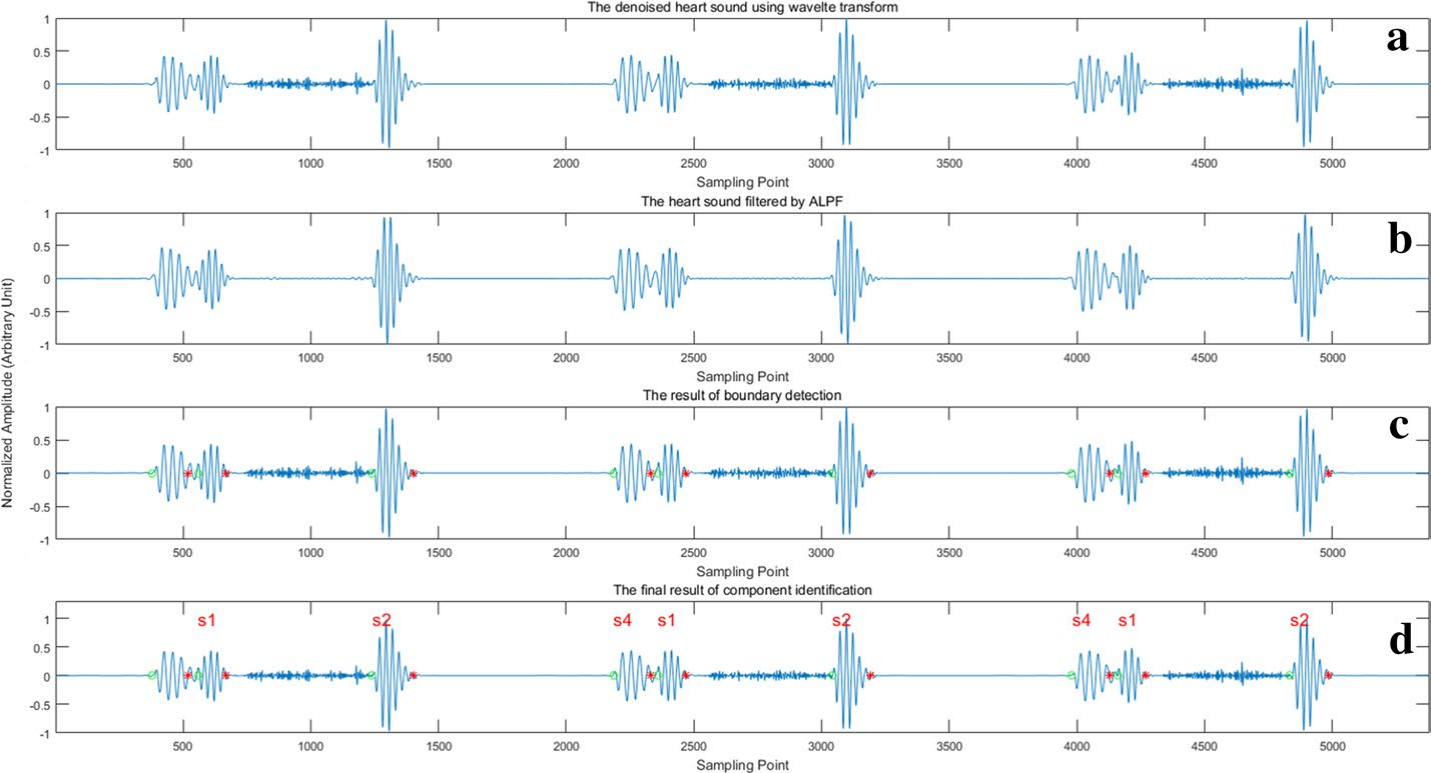
The results of murmur elimination, boundary detection and component identification for heart sound containing S4 and systolic murmurs

In this experiment, the onsets and offsets of heart sound components were automatically marked with green circles and red stars by the algorithm (see in Fig. 8c), respectively. In addition, the names of these components were automatically labeled above them (see in Fig. 8d). The results of boundary localization and component identification were judged by human and quantified using (17)–(19), in which the FN means false negative, FP means false positive, TN means true negative and TP means true positive.

### Results of murmur elimination

The performance of ALPF was compared with two existing methods. The first method is the conventional low-pass filter with a constant cutoff frequency of 200 Hz (200-LPF), and the second method is the wavelet package transform method with a db10 mother wavelet and a scale of 5 (WPT) [14]. In the WPT method, the heart sound components were reconstructed by nodes (5, 0) to (5, 5) to remove interference components such as murmurs. The SMR of the heart sounds filtered by the ALPF, WPT and 200-LPF are denoted as *SMR*_*A*_, *SMR*_*WPT*_, and *SMR*_200_, respectively. Moreover, the SMR of the original heart sounds *HS*_*N*_(*n*) was calculated and denoted as $SMR_{Ori}$. In addition, the SMR gains of these three methods were achieved by subtracting *SMR*_*Ori*_ from $SMR_{A}$, $SMR_{WPT}$ and $SMR_{200}$, respectively. All the experimental parameters of these three methods were identical.

Tables 1 and 2 show the statistical results of SMR and SMR gains. It is observed that the proposed ALPF has the highest score of SMR and SMR gains. The overall performances were evaluated by two indices, arithmetic average (AA) and weighted average (WA). AA was achieved by simply averaging the SMRs of 12 types of heart sounds and WA was affected by the weight of each type’s cycle number. In terms of SMR, the AA (± SD) and WA (± SD) of *SMR*_*A*_ reach 26.95 ± 4.30 db and 26.92 ± 4.14 db, respectively; by contrast, the AA and WA are 24.42.07 ± 4.54 and 24.38 ± 4.43 for *SMR*_*WPT*_ and 22.61 ± 5.86 and 22.61 ± 5.29 for *SMR*_200_. In terms of SMR gains, the heart sounds filtered by the ALPF have an arithmetic average and weighted average SMR gain (± SD) of 8.66 ± 4.52 db and 8.59 ± 4.37 db; by contrast, the arithmetic average and weighted average SMR gains (± SD) are 6.07 ± 3.88 and 5.99 ± 3.76 for WPT and 4.26 ± 4.51 and 4.21 ± 4.51 for 200-LPF.

**Table 1:** Statistics of SMR

UMich no.	Heart sound types	Length (s)	Cycle number	*SMR*_*A*_ */db*	SMR_WPT_/db	*SMR*_200_/db	*SMR*_*Ori*_/db
6	Early Systolic Murmur, Apex	60	66	*24.24*	22.91	15.52	14.60
7	Mid Systolic Murmur, Apex	67	72	*28.34*	25.37	22.01	12.51
8	Late Systolic Murmur, Apex	61	63	*30.27*	28.93	29.16	21.64
9	Holosystolic Murmur, Apex	60	63	*24.26*	22.20	21.96	16.07
4	Mid Systolic Click, Apex	60	72	*25.12*	22.25	19.50	19.14
10	Systolic Click and Late Systolic Murmur, Apex	64	72	*29.03*	26.35	24.18	23.23
11	S4 and Mid Systolic Murmur, Apex	65	72	*34.35*	28.18	28.93	17.06
12	S3 and Holosystolic Murmur, Apex	64	87	*27.63*	26.35	26.77	16.09
16	Early Diastolic Murmur, Aortic	61	81	*32.12*	31.92	31.88	31.37
21	Ejection Systolic Murmur and Transient Split S2, Pulmonic	56	75	*26.41*	23.68	21.33	20.47
22	Split S2 and Ejection Systolic Murmur, Pulmonic	66	78	*18.07*	14.46	12.52	11.26
23	Ejection Systolic Murmur and Single S2 and Ejection Click, Pulmonic	75	87	*24.30*	20.20	21.96	16.07
Arithmetic average ± SD				*26.95 ± 4.30*	24.42 ± 4.54	22.61 ± 5.86	18.35 ± 5.41
Weighted average ± SD				*26.92 ± 4.14*	24.38 ± 4.43	22.61 ± 5.67	18.39 ± 5.29

The italic values are represent the optimal results, compared with the results achieved in other methods/studies

**Table 2:** Statistics of SMR gains

UMich no.	Heart sound types	Length (s)	Cycle number	SMR gains		
				ALPF	WPT	200LPF
6	Early Systolic Murmur, Apex	60	66	*9.64*	8.31	0.91
7	Mid Systolic Murmur, Apex	67	72	*15.84*	12.86	9.50
8	Late Systolic Murmur, Apex	61	63	*8.64*	7.29	7.52
9	Holosystolic Murmur, Apex	60	63	*8.20*	6.13	5.90
4	Mid Systolic Click, Apex	60	72	*5.99*	3.11	0.37
10	Systolic Click and Late Systolic Murmur, Apex	64	72	*5.79*	3.12	0.95
11	S4 and Mid Systolic Murmur, Apex	65	72	*17.29*	11.12	11.88
12	S3 and Holosystolic Murmur, Apex	64	87	*11.54*	10.25	10.68
16	Early Diastolic Murmur, Aortic	61	81	*0.765*	0.55	0.51
21	Ejection Systolic Murmur and Transient Split S2, Pulmonic	56	75	*5.94*	3.21	0.86
22	Split S2 and Ejection Systolic Murmur, Pulmonic	66	78	*6.81*	3.20	1.26
23	Ejection Systolic Murmur and Single S2 and Ejection Click, Pulmonic	75	87	*7.51*	3.70	0.79
Arithmetic Average ± SD				*8.66 ± 4.52*	6.07 ± 3.88	4.26 ± 4.51
Weighted Average ± SD				*8.59 ± 4.37*	5.99 ± 3.76	4.21 ± 4.37

The italic values are represent the optimal results, compared with the results achieved in other methods/studies

Figure 7 shows an example of the murmur elimination results using the three methods.

### Results of boundary detection and component identification

A detailed quantitative description of the boundary positioning result is shown in Table 3. It is observed that the proposed method achieves a sensitivity of 100% for all 16 types of heart sounds, and both the PPV and accuracy are 99.93%, indicating an outstanding performance.

**Table 3:** Quantitative estimation of positioning

UMich no.	Heart sound types	Length (s)	Cycle number	TP	FN	FP	TN	Se/ %	PPV/ %	Acc/ %
1	Normal S1 and S2, Apex	69	87	174	0	0	0	100	100	100
2	Split S1, Apex	71	102	204	0	0	0	100	100	100
3	S4 Gallop, Apex	75	93	279	0	0	0	100	100	100
4	Mid Systolic Click, Apex	60	72	144	0	2	0	100	98.63	98.63
5	S3 Gallop, Apex	68	81	243	0	0	0	100	100	100
6	Early Systolic Murmur, Apex	60	66	132	0	0	0	100	100	100
7	Mid Systolic Murmur, Apex	67	72	144	0	0	0	100	100	100
8	Late Systolic Murmur, Apex	61	63	126	0	0	0	100	100	100
9	Holosystolic Murmur, Apex	60	63	126	0	0	0	100	100	100
10	Systolic Click and Late Systolic Murmur, Apex	61	72	144	0	0	0	100	100	100
11	S4 and Mid Systolic Murmur, Apex	65	87	261	0	0	0	100	100	100
12	S3 and Holosystolic Murmur, Apex	64	87	261	0	0	0	100	100	100
14	Normal S1 and S2, Aortic	61	69	138	0	0	0	100	100	100
16	Early Diastolic Murmur, Aortic	61	81	162	0	0	0	100	100	100
18	Single S2, Pulmonic	61	69	138	0	0	0	100	100	100
23	Ejection Systolic Murmur and Single S2 and Ejection Click, Pulmonic	75	87	174	0	0	0	100	100	100
Total		1039	1251	2850	0	2	0	100	99.93	99.93

Table 4 presents the quantitative data of component identification. Because of the good positioning effect, the identification performance is high, with an average Se of 98.63%, an average PPV of 99.86%, and an average accuracy of 98.49%.

**Table 4:** Quantitative estimation of identification

UMich no.	Heart sound types	Length (s)	Cycle number	TP	FN	FP	TN	Se/ %	PPV/ %	Acc/ %
1	Normal S1 and S2, Apex	69	87	174	0	0	0	100	100	100
2	Split S1, Apex	71	102	204	0	0	0	100	100	100
3	S4 Gallop, Apex	75	93	279	0	0	0	100	100	100
4	Mid Systolic Click, Apex	60	72	142	3	4	0	97.73	97.26	95.30
5	S3 Gallop, Apex	68	81	243	0	0	0	100	100	100
6	Early Systolic Murmur, Apex	60	66	132	0	0	0	100	100	100
7	Mid Systolic Murmur, Apex	67	72	144	0	0	0	100	100	100
8	Late Systolic Murmur, Apex	61	63	126	0	0	0	100	100	100
9	Holosystolic Murmur, Apex	60	63	126	0	0	0	100	100	100
10	Systolic Click and Late Systolic Murmur, Apex	61	72	144	0	0	0	100	100	100
11	S4 and Mid Systolic Murmur, Apex	65	87	261	0	0	0	100	100	100
12	S3 and Holosystolic Murmur, Apex	64	87	261	0	0	0	100	100	100
14	Normal S1 and S2, Aortic	61	69	102	36	0	0	73.91	100	73.91
16	Early Diastolic Murmur, Aortic	61	81	162	0	0	0	100	100	100
18	Single S2, Pulmonic	61	69	138	0	0	0	100	100	100
23	Ejection Systolic Murmur and Single S2 and Ejection Click, Pulmonic	75	87	174	0	0	0	100	100	100
Total		1039	1251	2812	39	4	0	98.63	99.86	98.49

Figure 8 shows the complete process of the proposed method, including murmur elimination, boundary detection, and component identification. The first component (S4) in Fig. 8d is not labeled. This is because the identification of S4 requires information obtained from the previous cardiac cycle. However, this cycle lacks the information. The unlabeled S4 component is identified in the previous segment. Therefore, there is no problem of missed detection.

## Discussion

Tables 1 and 2 show the results of murmur elimination, all three methods exhibit an improvement in SMR compared with *SMR*_*Ori*_. As a result of selecting the appropriate cutoff frequency, the proposed ALPF has the highest SMR gain among all the tested methods. The arithmetic and weighted average SMR gains of ALPF reach 8.66db and 8.59db, which are 42.67% and 43.41% higher than WPT’s 6.07db and 5.99db, and 103.29% and 104.04% higher than 200-LPF’s 4.26db and 4.21db, respectively, indicating optimal murmur elimination performance.

In order to verify the superiority of the proposed method for murmur elimination, in this study, the paired two-sample t-test was performed on the SMR gains calculated for 12 types of heart sounds filtered with these three methods. In the paired two-sample t-test, the significance level α was set to 0.01. Under this condition, the rejection region is |*t*| ≥ *t*_*α*/2_(*n* − 1) = *t*_0.005_(11) = 3.1058, and *t*_*A*,*WPT*_ = 5.5499 > 3.1058, *t*_*A*,200_ = 5.5039 > 3.1058, and *t*_*WPT*,200_ = 2.6273 < 3.1058. Thus, the proposed murmur elimination method was verified to have significant advantages compared with WPT and 200-LPF.

For boundary detection results shown in Table 3, among all 16 types of heart sounds, the proposed method failed on one signal labeled as a mid-systolic click: two click components were not removed in boundary detection process and were erroneously positioned as true components. Time–frequency analysis was then performed to determine the reason for this.

The time–frequency analysis revealed that these click components have a relatively high number of overlaps with S1 and S2 in frequency, resulting in the failure in removing them. Compared with this sound, the other signals’ interference components (clicks, murmurs, and residues of noise) show a smaller overlap with the true components; thus, the positioning score is higher. In fact, setting a higher threshold *θ* can completely remove the clicks, resulting in a higher performance score. However, the higher threshold causes a larger deviation between the calculated boundaries and the real values. After weighing the pros and cons, this approach was abandoned.

The identification results of Table 4 shows that the fourth signal (Umich No. 4) is consistent with the result of positioning. A difference appears in the result of the 13th signal (Umich No. 14). The FN of the signal reaches 36, resulting in a low Se and low Acc of 73.91%. Initially, this result was puzzling because the signal was labeled as normal. After a comprehensive analysis, the rhythm of some parts of this heart sound was found to be irregular, resulting in the failure of heart cycle calculation in these parts. Therefore, components of these parts could not be recognized.

For the performance evaluation, because the cycle number of each heart sound was very close and the performance of each type of signal was analyzed separately, the utilized dataset did not suffer from the problem of unbalance.

Some other operating characteristics obtained in this study and 11 previous studies are listed in Table 5 for comparison. Both the Se and PPV for positioning in literature [6] consist of two values separated by a slash. The two values are for normal and pathological cases of heart sounds, respectively. As mentioned in the “Background”, a complete segmentation is comprised of boundary detection and component identification. However, several existing works only focus on one of them. Moreover, some studies could be applied only to the basic situation, for example, heart sounds without murmurs. Therefore, in addition to achieving excellent performance, the proposed approach achieves the functions of both boundary localization and component identification and can be applied in more conditions.

**Table 5:** A comprehensive comparison with other studies

Methods	Application capability^a^				Functions^b^		Positioning^c^			Identifying		
	N	S3	S4	Mur	P	I	Se	PPV	Acc	Se	PPV	Acc
Proposed method	✓	✓	✓	✓	✓	✓	*100*	*99.93*	*99.93*	98.63	*99.86*	*98.49*
Naseri et al. [8]	✓	✓	✓	✓	✗	✓	✗	✗	✗	*99.00*	98.60	NM
Varghees et al. [21]	✓	✓	✓	✓	✓	✗	99.43	93.56	93.06	✗	✗	✗
Sepehria et al. [22]	✓	✓	✓	✓	✗	✓^*^	✗	✗	✗	✗	✗	93.6
Moukadem et al. [6]	✓	✗	✗	✓	✓	✓	96/97	95/95	NM	95	97	NM
Moukadem et al. [26]	✓	✗	✗	✓	✓	✗	95	98	NM	✗	✗	✗
Pedrosa et al. [23]	✓	✗	✗	✗	✓	✓	89.2	98.6	NM	✗	✗	✗
Wang et al. [24]	✓	✗	✗	✓	✓	✓	NM	NM	NM	NM	NM	NM
Schmidt et al. [25]	✓	✗	✗	✗	✗	✓	✗	✗	✗	98.8	98.6	NM
Tseng et al. [2]	✓	✗	✗	✗	✗	✓	NM	NM	NM	92.4	88.1	NM
Zhong et al. [27]	✓	✗	✗	✗	✓	✓	NM	NM	NM	92.84	NM	NM
Wang et al. [28]	✓	✗	✗	✗	✓	✓	NM	NM	NM	96.8	NM	NM

The italic values are represent the optimal results, compared with the results achieved in other methods/studies

^a^N: normal; Mur: murmurs

^b^P: positioning; I: identifying; ✓^*^: only available for S1 and S2

^c^NM: not mentioned

## Conclusions

This paper presents an accurate heart sound segmentation algorithm that combines time-domain, frequency-domain and time–frequency-domain analysis. Compared to existing studies, this method is applicable to a wide range of heart sounds, from normal to those containing S3, S4 and various murmurs. To verify this method, quantitative experiments were performed using the University of Michigan’s Heart Sound & Murmur Library, an authoritative open database. The experimental materials incorporated two types of normal heart sounds and 14 types of abnormal heart sounds. The results show that the boundary localization has an average Se of 100%, an average PPV of 99.3% and an average Acc of 99.93%. Moreover, the Se, PPV and Acc of the component identification reach 98.63%, 99.86% and 98.49%, respectively, indicating outstanding performance of the proposed method. There are still some shortcomings of this work. For example, the component identification relies on the success of cardiac cycle calculation; therefore, this method cannot be applied to the heart sound with severe arrhythmia because of the failure to achieving accurate cardiac cycle by using UACF. The study of segmentation provides a good basis for extracting significant features of heart sounds. Therefore, the further study will focus on the classification of heart sounds.
